# Effects of Acupuncture on Behavioral Stereotypies and Brain Dopamine System in Mice as a Model of Tourette Syndrome

**DOI:** 10.3389/fnbeh.2019.00239

**Published:** 2019-10-15

**Authors:** Lixue Lin, Lingling Yu, Hongchun Xiang, Xuefei Hu, Xiaocui Yuan, He Zhu, Hongping Li, Hong Zhang, Tengfei Hou, Jie Cao, Shuang Wu, Wen Su, Man Li

**Affiliations:** ^1^Department of Neurobiology, School of Basic Medicine, Tongji Medical College, Huazhong University of Science and Technology, Wuhan, China; ^2^Department of Neurology, Tongji Hospital, Tongji Medical College, Huazhong University of Science and Technology, Wuhan, China; ^3^Department of Pediatrics, Wuhan No. 1 Hospital, Wuhan, China

**Keywords:** Tourette syndrome, acupuncture, dopamine system, striatum, substantia nigra pars compacta, prefrontal cortex

## Abstract

Tourette syndrome (TS), a developmental neurobehavioral disorder, is characterized by involuntary behavioral stereotypies. Clinical studies have confirmed the positive effect of acupuncture on treating TS, but the underlying mechanisms are not fully understood. In the present study, we used behavioral tests, Western blotting, double-immunofluorescence labeling, and fluorescence spectrophotometry to investigate whether acupuncture performed at acupoints “Baihui” (GV20) and “Yintang” (GV29) affected behavioral stereotypies and regulated the dopamine (DA) system in three different brain regions in Balb/c mice injected with 3,3′-iminodipropionitrile (IDPN) as a model for TS. We found that acupuncture alleviated behavioral stereotypies, down-regulated the expression of D1R and D2R in the striatum (STR) and substantia nigra pars compacta (SNpc), and decreased the concentration of DA in the STR, SNpc, and prefrontal cortex (PFC) as well. Moreover, acupuncture reduced the expression of tyrosine hydroxylase (TH) in the SNpc. Conclusively, acupuncture ameliorated behavioral stereotypies by regulating the DA system in the STR, SNpc, and PFC. Our findings provide novel evidence for the therapeutic effect of acupuncture on TS.

## Introduction

Tourette syndrome (TS) is a neurodevelopmental disorder in children, which is characterized by multiple motor and behavioral stereotypies. The main presentations of behavioral stereotypies are involuntary, quick, sudden, and stereotypical movements or phonic productions (Kawohl et al., 2009). Common complications of TS include attention-deficit/hyperactivity disorder, obsessive–compulsive disorder, and learning disability. The rate of incidence of TS is increasing every year, a trend that seriously reduces the quality of life and affects the society as a whole (Du et al., 2010). TS is highly heritable but no specific genes have been strongly implicated in TS and the pathophysiology remains unknown (Wang et al., 2007).

In recent years, multiple functional magnetic resonance imaging (fMRI) studies have shown several structural abnormalities in the brain of TS patients (Cui et al., 2014; Deckersbach et al., 2014; Tinaz et al., 2014; Eddy et al., 2016). Either deficient frontal inhibition for structural changes or desynchronization in the interaction of the cortico-striato -thalamo-cortical network within the limbic system could be the main pathogenesis of TS (Neuner et al., 2010; Singer, 2013; Cui et al., 2014; Deckersbach et al., 2014; Tinaz et al., 2014; Eddy et al., 2016). The severity of the symptoms is closely associated with the hyperfunction of the nigrostriatal dopaminergic neurons (Cui et al., 2013; Zebardast et al., 2013). Furthermore, different neurotransmitters can activate or suppress striatal activity. Over the years, the “DA hypothesis” of TS was prevalent (Butler et al., 1979; Singer et al., 1982). It proposed that the hypersensitivity of DA receptors and the hyperactivity of dopaminergic neurons in nigrostriatum underlay the pathophysiology of TS (Steeves et al., 2010), including increased production of DA and its metabolites (Wang et al., 2012), and supersensitivity of dopaminergic receptors (Singer et al., 1982). Dopamine (DA) is a neurotransmitter that can regulate neural processes including motor control, cognition, and memory (Chun et al., 2013). Previous studies have suggested that the imbalanced expression of DA plays an important role in the development of TS (Nespoli et al., 2018).

Dopamine beta hydroxylase catalyzes the conversion of DA to norepinephrine in the adrenal medulla (Kirshner, 1957). Tyrosine hydroxylase (TH) is the rate-limiting enzyme in catecholamine biosynthesis in the brain and is responsible for the conversion of L-tyrosine to 3,4 dihydrophenylalanine (DOPA) (Brett et al., 1995; Zhu et al., 2012; Tekin et al., 2014). Dysregulation of TH activity is essential in the pathogenesis of TS (Brett et al., 1995; Comings et al., 1995; Qi et al., 2018).

The striatal disinhibition model of TS provides a unique combination of face validity (tic expression) and construct validity (abnormal striatal inhibition) but is limited to sub-hour periods (Bronfeld et al., 2013; Israelashvili and Bar-Gad, 2015). Other TS model mice are established by injecting GABA_A_ antagonists into the STR, leading to temporary disinhibition (Vinner et al., 2017). Although the tic symptom of this model was stable, its modeling method is very complicated. IDPN is a synthetic nitrile that causes dyskinesia and persistent behavioral syndromes characterized by repetitive head movements, circling, hyperactivity, and retropulsion (Khan and Ibrahim, 2015). Some reports show that these behavioral abnormalities are caused by monoamine changes (Nomoto, 2004; Guo and Ou-Yang, 2008). It is a widely used animal model simulating behavioral stereotypies of TS (Wang et al., 2013; Zhang et al., 2014; Zhang and Li, 2015a,b,c; Zhao et al., 2015; Xie et al., 2016). IDPN-induced dyskinesia is enhanced by levodopa, which increases the DA concentration in the nucleus of the accumbens and midbrain. The increase of DA is inhibited by sulpiride, a central antagonist of D2R. Therefore, dyskinesia of DA is caused by the abnormally enhanced function of presynaptic DA neurons (Ogawa et al., 1990). IDPN only needs to be injected intraperitoneally, which makes it easy to prepare and utilize, so we used IDPN to induce a model of TS in mice.

At present, there are no safe and effective drugs for treating TS. In clinical practice, the drugs used in the treatment of TS are mainly DA receptor antagonists (haloperidol, pimozide, and tiapride) (Cath et al., 1999; Parraga et al., 2010; Buse et al., 2013; Tang et al., 2015; Vicario et al., 2016; Roth, 2018), selective monoaminergic antagonists (clozapine and risperidone) (Piccinni et al., 2013; Roessner et al., 2013; Egolf and Coffey, 2014), and α-2 adrenergic agonists (clonidine) (Cavanna et al., 2012; Eapen et al., 2014). However, these drugs have toxic side effects. Acupuncture is a unique and complete treatment in traditional Chinese medicine and has been practiced in China for thousands of years (Faircloth, 2015; Mallory et al., 2016; Ren, 2017). The effectiveness and safety of acupuncture for TS has been confirmed by numerous clinical studies (Wu et al., 1996; Ma et al., 2006; Zhu et al., 2009; Tang et al., 2015; Yu et al., 2016; Lee, 2017), but the underlying mechanisms remain unclear. Previous studies have demonstrated that acupuncture can regulate the DA system (Bo et al., 2017; Lin et al., 2017; Kim et al., 2018; Xiao et al., 2018). DA plays an important role in the recovering effect of acupuncture on motor functions (Cabyoglu et al., 2006). For example, acupuncture at a specific acupoint “Shenmen” (HT7) reduces DA release in the nucleus accumbens (NAc) induced by cocaine abuse (Jin et al., 2018). Meanwhile, acupuncture regulates the levels of neurotransmitters such as DA and serotonin, thereby affecting emotional state and craving; this is the basis for the treatment of smoking addiction with acupuncture (Cheng, 2014). Thus, we hypothesize that acupuncture may regulate the DA system in mice injected with 3,3′-iminodipropionitrile (IDPN) as a model for TS.

Therefore, we wondered whether acupuncture alleviated behavioral stereotypies by regulating the production of DA and its receptors in different brain regions in mice injected with IDPN as a model for TS. In the present study, we first evaluated the changes of behavioral stereotypies after acupuncture applied at acupoints “Baihui” (GV20) and “Yintang” (GV29) in mice injected with IDPN as a model for TS (Khan and Ibrahim, 2015; Tang et al., 2015). We then determined whether DA, D1 receptor (D1R), D2 receptor (D2R), and TH in the striatum (STR), substantia nigra pars compacta (SNpc), thalamus, and prefrontal cortex (PFC) were involved in the mechanism of acupuncture alleviating IDPN-induced behavioral stereotypies.

## Materials and Methods

### Animal Models

All Balb/c mice (male, aged 8 weeks, and 18–21 g) were purchased from Vital River Laboratories (Beijing, China). All animal experimental protocols conformed to the Animal Management Rules of the Chinese Ministry of Health, and the study was approved by the animal ethics committee of the Chinese Academy of Medical Sciences. The mice were maintained in a controlled environment with temperature at 21 ± 1°C, relative humidity of 60 ± 10% under a 12-h light/dark cycle (lights on at 7 a.m.) and had free access to food and water. The mice were housed individually in standard PP polypropylene plastic cages (318 × 202 × 136 mm) with sawdust bedding and water and food *ad libitum* (Xietong Pharmaceutical Biotechnology Limited Liability Company, Jiangsu, China).

The adaptation period between arrival at the laboratory and start of testing was 1 week. After 1 week, mice were randomly divided into the control group, IDPN-induced TS model group (IDPN group), IDPN-induced TS model with acupuncture with twisting group (acupuncture group), IDPN-induced TS model with acupuncture without twisting group (sham acupuncture 1 group), and IDPN-induced TS model with acupuncture on non-acupoints with twisting group (sham acupuncture 2 group). Each group had 18 mice. The control group was intraperitoneally injected (i.p.) with saline (0.9%); the IDPN group, acupuncture group, and two sham acupuncture groups were intraperitoneally injected with IDPN (350 mg/kg, Sigma, St. Louis, MO, United States) once a day for seven consecutive days (days 1–7). The ethological score between each group was balanced, referring to the evaluating grade of stereotypy (Table 1; Diamond et al., 1982; Khan et al., 2009). On the eighth day, the stereotyped behavior scores of the IDPN group, acupuncture group, and two sham acupuncture groups were greater than or equal to 2 points, which proved successful IDPN-induced TS model.

**TABLE 1:** Behavior measurements referring to evaluating grades of stereotypy.

**Score**	**Stereotypy**
0	No stereotypy or normal activity^a^
1	Discontinuous circling behavior^b^ Occasional head twitching
2	Occasionally vertical dyskinetic head and neck movements Occasional sniffing, licking, and biting
3	Continuous circling behavior, increased body raising Increased sniffing, repetitive grooming (such as paw-to-mouth movements)^c^
4	Increased lateral and vertical dyskinetic head and neck movements

*^*a*^Such as immobility (no visible movement of the animal); ^*b*^Such as clockwise/counterclockwise circling, loosely circular path traced on the cage floor; ^*c*^Such as head-down sniffing (nose in contact with the floor), grooming (self-cleaning with mouth or paws), face washing (forepaws moving back and forth from the ears to the snout and mouth).*

### Acupuncture Treatment

In the acupuncture group and sham acupuncture group, the mice were treated by acupuncture once every other day for 8–36 days. In the acupuncture group, acupuncture was applied at acupoints “Baihui” (GV20) and “Yintang” (GV29). GV20 and GV29 were chosen based on their effect in TS (Tang et al., 2015). Two acupuncture needles (0.18 × 13 mm) were inserted 2–3 mm into two acupoints of GV20 and GV29, and manually rotated for 20 s every 5 min (twisting speed 100–200 times/min), 30 min each time. GV20 lies on the back of the hairline and is 7 inches in the middle; when the two ears are straight up, the top of the head is in the middle. GV29 is located at the forehead, where the line between the brows and anterior midline intersects (Fang, 1993) (Supplementary Figure S1C). Our therapist is trained after acupuncture techniques, plus continuous practice of manual rotation. In the acupuncture group and sham acupuncture 2 group, each mouse was videotaped with a camera during acupuncture treatment. Then, we let the three observers watch the playback video and counted it with a counter. The number of twisting times obtained by three observers was averaged. The average was 100–200 times, and we think that manual rotation is eligible.

The treatment time of the two sham acupuncture groups was the same as that of the acupuncture group, but needles were not manually twisted in the sham acupuncture 1 group. In the sham acupuncture 2 group, needles were inserted 2 mm lateral to GV20 and GV29, respectively, and needles were manually twisted with the same speed.

During acupuncture or sham acupuncture treatment, each mouse was placed in a homemade bag but not given any anesthetics, and they were not in contact with the experimenter. The homemade clothes were made with a piece of 10 × 10 cm jean. Limbs of mouse were pulled out through the holes in the clothes. The edge of the clothes was fastened by clips (Supplementary Figure S1D). The control group and IDPN group also used the same homemade bag to exclude the stress response. The animals remained awake during treatment and showed no evident signs of distress.

### Behavioral Tests

All behavioral tests were carried out once every week on days 0–36 after intraperitoneal injection or 1 h after acupuncture treatment (Supplementary Figure S1A). First, we performed the stereotyped behavior test, then we performed the rotarod test, and finally we performed the grip strength test. Before the behavioral tests, the animals were habituated to the testing environment for 30 min. The experimenters of the behavioral tests randomly tested all mice according to the random number table method.

#### Stereotyped Behavior Test

The stereotyped behavior test was conducted by two trained and independent observers, who were familiar to the measurements but blinded to the group allocation. They grouped the mice themselves and then randomly tested all mice and scored them according to the random number table method. Finally, the experiment results were correctly counted by an experimenter who knew the group allocation. The observers placed the mice in the box, and then recorded a 30-min video for each mouse. The box used for stereotyped behavior was a round black plastic box with a diameter of 16 cm and a height of 14 cm, and the camera was placed at the top of the box (Supplementary Figure S1E). After each test of one mouse, the observers sprayed the inside and bottom of the box and the transparent lid with 75% alcohol, and wiped it with a paper towel before placing the next mouse in it. The light condition during the observation period was the LED tube (consistent with the illumination of the usual living environment of the mouse), and the light condition lasted throughout the experiment. Each animal was observed for 1 min of every 5 min for a total of six periods. One or more episodes that were in accordance with the grades got the corresponding score and calculated the average score on the basis of results from two observers, as the objective indicator of behavioral changes (Table 1; Wang et al., 2013; Proietti Onori et al., 2014).

#### Rotarod Test

To assess coordination and balance, mice were individually placed on the rotating rotarod (YLS-4C, Yiyan, Jinan, China) for 5 min at 4 r/min on 10:00 a.m. and 5:00 p.m. for 3 days before the formal test, in order to let the mice learn how to use. The rotarod moved at an initial speed of 4 r/min and subsequently accelerated to 40 r/min in 5 min. Mice were held by the tail and placed on the rotarod, facing away from the direction of rotation. The fall time after the beginning of the acceleration was recorded. Each mouse received three test sessions with at least a 5-min interval between them, and the average time was calculated (Duo et al., 2018).

#### Grip Strength Test

In order to evaluate the muscle strength of mice, we did the grip strength test in mice. We followed the manufacturer’s instruction for the grip Strength Meter (YLS-13A, Zhenghua, Anhui, China) and the statistical indicator was the grip force (*g*). Mice were placed over a base plate (230 mm × 250 mm), in front of a grasping bar. The bar was fitted to a force transducer connected to the peak amplifier. Mice were pulled by the tail when grasping the bar. Within 20 s, the maximal grip force was measured (Huang et al., 2014).

### Western Blotting

On the 37th day, the STR, SNpc, thalamus, and PFC tissues were excised from mice immediately after the mice were anesthetized with 1% sodium pentobarbital anesthesia (50 mg/kg, i.p.) and decapitated (*n* = 6 in each group). The tissues were homogenized in RIPA lysis buffer (40 mg/ml for tissues, Beyotime Biotechnology, Nanjing, China) and 2 mM phenylmethylsulfonyl fluoride, put on ice for 30 min, and centrifuged at 12,000 *g* for 15 min at 4°C. Then, we took the supernatant and discarded the pellet. The protein concentrations of the supernatant were determined using the Enhanced BCA Protein Assay Kit (Beyotime Biotechnology, China). Each sample was denatured with loading buffer at 95°C for 5 min, and separated with a 10–12% glycine SDS-PAGE (sodium dodecyl sulfate polyacrylamide gel electrophoresis) gel. The proteins were transferred onto a polyvinylidene fluoride (PVDF) membrane, and blocked for 1 h in 5% non-fat milk in Tris-buffered saline (TBS) containing 0.1% Tween-20 on a shaker at room temperature. The membrane was incubated with rabbit anti-D1R antibody (1:1000, Abcam, Hong Kong), rabbit anti-D2R antibody (1:200, Cloud-Clone), rabbit anti-TH antibody (1:3000, Proteintech), and mouse anti-GAPDH antibody (1:10000, Proteintech) on a shaker at 4°C overnight. After washing three times for 10 min in 0.1% TBS–Tween 20 (pH 7.4) at room temperature, the membrane was then incubated with horseradish peroxidase-conjugated secondary antibodies from Santa Cruz Biotechnology: goat anti-rabbit secondary antibody (1:20,000) or goat anti-mouse secondary antibody (1:20,000) for 1 h on a shaker at room temperature, and washed three times for 10 min with 0.1% TBS–Tween 20 (pH 7.4) on a shaker. The enhanced chemiluminescence method (ECL Plus Western blotting detection reagents, Pierce, Rockford, IL, United States) was used to visualize the protein bands. Image Lab software was used for imaging on a computer-assisted imaging analysis system (Quantity One, Bio-Rad, United Kingdom). The optical density of each band was then measured with an imaging analysis system and normalized with the housekeeping gene GAPDH. Results of independent experiments were expressed as the% change over the protein amount in the control group. Densitometry analysis of the gel images was performed using the ImageJ software (NIH, Bethesda, MD, United States). The layouts of gel images were based on the GraphPad prism 5.01 (GraphPad Company, United States).

### Double-Immunofluorescence Labeling

On the 37th day, 30 mice (*n* = 6 in each group) were deeply anesthetized with 1% sodium pentobarbital anesthesia (50 mg/kg, i.p.) and transcardially perfused with 100 ml of 37°C normal saline followed by 50 ml of 4% paraformaldehyde in 0.1 M phosphate buffer (PBS, pH 7.4) at 4°C for fixing. The brain tissues were quickly separated and post-fixed for 6–8 h in the same fixative solution and dehydrated in 20% sucrose in 0.1 M PBS for 24 h and 30% sucrose in 0.1 M PBS for 24 h at 4°C. The sections were cut (30 μm in thickness) on a cryostat, mounted onto gelatin-coated slides, and air-dried overnight (Wu et al., 2016).

The sections were rinsed in 0.01 M PBS and blocked for 1 h with 5% donkey serum and 0.2% Tween-20 in PBS and then incubated with the following primary antibodies at 37°C for 1 h and at 4°C overnight: rabbit anti-D1R (1:500, Abcam), rabbit anti-D2R (1:100, Cloud-Clone), rabbit anti-TH (1:100, Proteintech), and mouse anti-NeuN (1:100, Servicebio). Subsequently, sections were washed four times in PBS for 5 min and incubated with corresponding secondary antibodies from Jackson Immune Research (West Grove, PA, United States): donkey anti-rabbit IgG conjugated with DyLight 488 (1:400) and donkey anti-mouse IgG conjugated with DyLight 594 (1:400). Then, the sections were incubated with DAPI for the cell nucleus staining for 8 min. Sections were washed four times with 0.05% Tween-20 in PBS for 5 min and then cover slipped. An Olympus BX51 fluorescence microscope was used to view the sections, and images were captured using a Qimaging Camera and QCapture software. A total of five to six sections were randomly selected in each mouse. Images were analyzed by using NIH Image J software (Bethesda, MD, United States). The layouts of images were based on the Photoshop CS5 (ADOBE Company, United States).

### Measurements of DA

Fluorescence spectrophotometry was used because of its advantages of high selectivity, high sensitivity, low detection limit, wide application, and abundant information (Yuan et al., 2018). After sacrificing the mouse, brain tissue was immediately removed (*n* = 6 in each group). After weighing, specimens were homogenized in 3 ml of acid butyl alcohol and centrifuged at 3000 *g* for 5 min, and then the supernatant was collected. The supernatant (0.4 ml) was added to 0.8 ml of n-heptane and 0.16 ml of 0.1 N HCl, vortexed for 5 min, and centrifuged at 3000 *g* for 5 min at room temperature. The water phase (256 μl) was collected and mixed with 1.025 ml of 0.1 mol/L sodium tetraborate buffer, 51 μl of 0.002% CuCl_2_, 51 μl of 0.25% potassium ferricyanide, 51 μl of 10% mercaptoethanol, 256 μl of 8 mol/L NaOH, and 102 μl of 10 mol/L glacial acetic acid. After mixing thoroughly, the solution was placed into boiling water for 10 min, until it was cooled to room temperature; the solution was added to 205 μl of 45% phosphate buffer and then placed into boiling water for 10 min. The fluorescence intensity of DA was measured at 410/270 nm by an F-4500 Fluorescence Spectrophotometer (Hitachi, Japan) and DA concentration was determined by comparing to a standard DA dilution (Li et al., 2003).

### Data Analysis

All data analysis was conducted on GraphPad Prism 7 (GraphPad Software, Inc., La Jolla, CA, United States). Data are presented as means ± SEM. We used one-way analysis of variance (ANOVA) or repeated-measure ANOVA to determine the overall effect of interventions. Tukey’s *post hoc* test was then used to determine the statistical difference in biochemical data between individual groups. To determine the statistical difference in behavioral data between different groups and time points, we used Bonferroni’s *post hoc* test. A *P* value of less than 0.05 was considered statistically significant.

## Results

### Acupuncture Alleviated Involuntary Behavioral Stereotypies of a Mouse Model of TS

**FIGURE 1:**
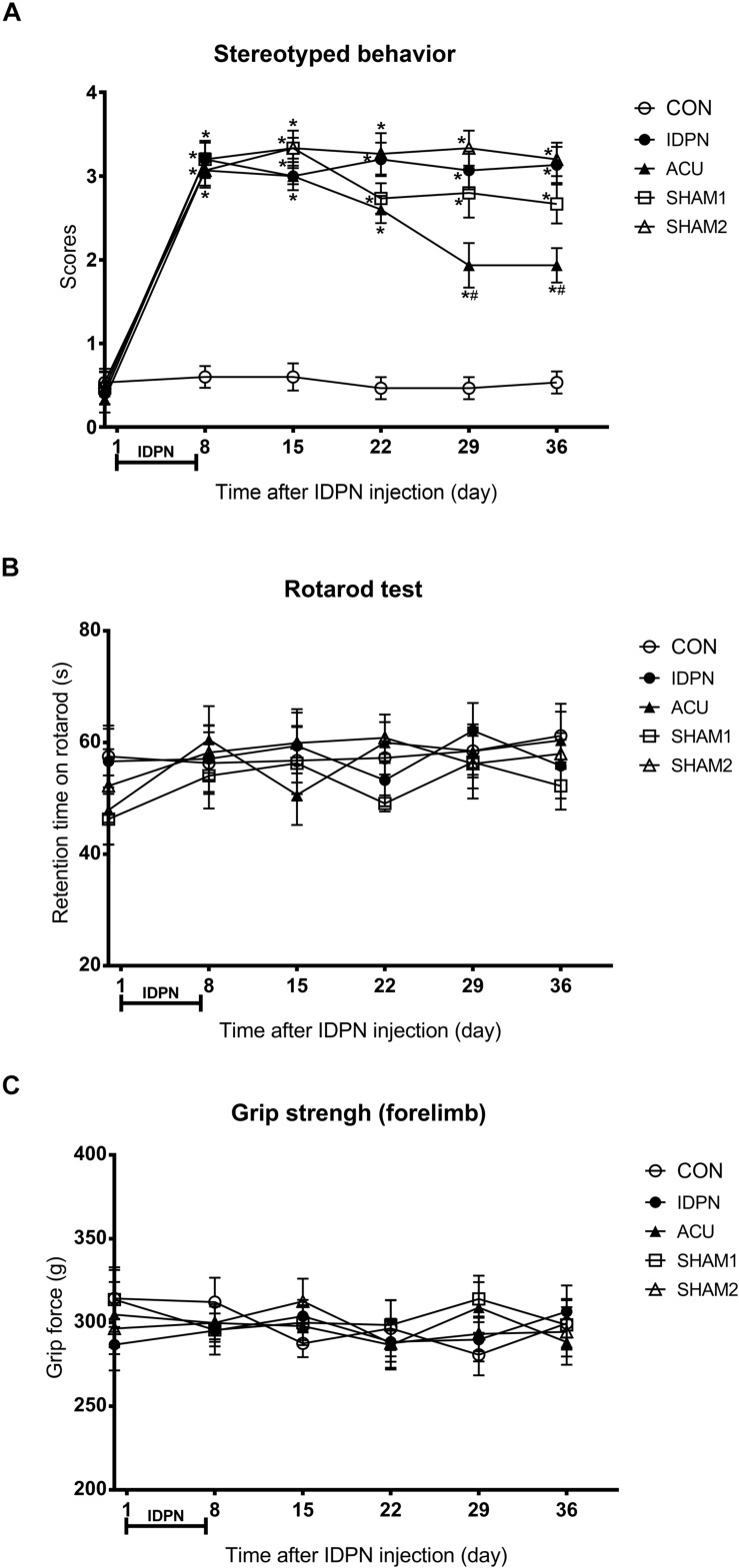
Effects of acupuncture on stereotyped behavior, motor coordination, and muscle strength in a mouse model of TS. **(A)** Evaluations of stereotyped behavior scores of mice in the control group (CON group), IDPN-induced TS model group (IDPN group), IDPN-induced TS model with acupuncture with twisting group (ACU group), IDPN-induced TS model with acupuncture without twisting group (SHAM1 group), and IDPN-induced TS model with acupuncture on non-acupoints with twisting group (SHAM2 group) on days 0, 8, 15, 22, 29, and 36. **(B)** Latency to fall in the rotarod test among the CON, IDPN, ACU, SHAM1, and SHAM2 groups on days 0, 8, 15, 22, 29, and 36. **(C)** Forelimb grip force among the CON, IDPN, ACU, SHAM1, and SHAM2 groups on days 0, 8, 15, 22, 29, and 36. Data are expressed as means ± SEM (*n* = 18 mice in each group). ^∗^*P* < 0.05, compared with the CON group; ^#^*P* < 0.05, compared with the IDPN group.

In order to explore the extent of involuntary behavioral stereotypies and changes of other related behaviors in a mouse model of TS, we performed a series of behavioral tests. On the eighth day, we found that the score of stereotyped behavior in the IDPN, acupuncture, and two sham acupuncture groups were significantly higher than that of the control group [score: *F*(4,420) = 146.7, *P* < 0.0001], indicating successful modeling. The scores of stereotyped behavior [time × score: *F*(20,420) = 8.149, *P* < 0.0001] of the acupuncture group significantly reduced after the 22nd day, and there was no significant difference in the two sham acupuncture groups and IDPN groups (Figure 1A). These data indicated that acupuncture alleviated the involuntary behavioral stereotypies and hyperactivity in the mouse model of TS.

In addition, the rotarod and the grip strength tests were used to assess the motor coordination ability and the muscle strength of the mice, respectively (Huang et al., 2014; Duo et al., 2018). However, IDPN, acupuncture, or sham acupuncture treatment had no influence on retention time on the rotarod [time × score: *F*(20,359) = 0.3865, *P* = 0.9929] and the grip force [time × score: *F*(20,420) = 0.4925, *P* = 0.9692], compared with the control group (Figures 1B,C). It indicated that IDPN or acupuncture did not affect the motor coordination ability and the muscle strength of a mouse model of TS. All animals gained weight during the test period, there was no significant difference in body weight between the control and IDPN groups (Supplementary Figure S1B).

### Acupuncture Decreased the Concentration of DA in the STR, SNpc, and PFC, and Down-Regulated TH Expression in the SNpc

**FIGURE 2:**
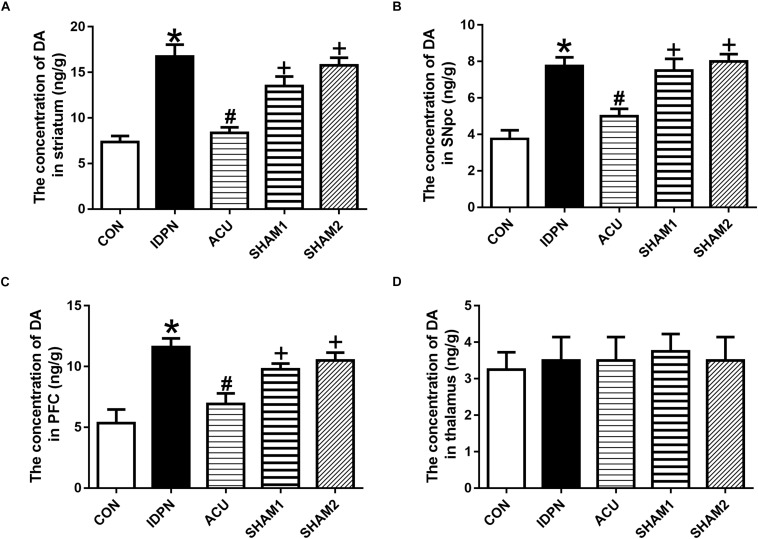
Effects of acupuncture on DA concentration in the STR, SNpc, and PFC. **(A)** Quantitative analysis of DA concentration in the STR of different groups. **(B)** Quantitative analysis of DA concentration in the SNpc of different groups. **(C)** Quantitative analysis of DA concentration in the PFC of different groups. **(D)** Quantitative analysis of DA concentration in the thalamus of different groups. Data are expressed as means ± SEM (*n* = 6 mice in each group). ^∗^*P* < 0.05, compared with the CON group; ^#^*P* < 0.05, compared with the IDPN group; ^+^*P* < 0.05, compared with the ACU group.

The concentration of DA is closely related to behavioral stereotypies (Wang et al., 2013). We determined whether acupuncture affected DA concentration in the STR, SNpc, PFC, and the thalamus. We found that IDPN significantly increased the level of DA in the STR, SNpc, and PFC but not in the thalamus, which was significantly reversed by acupuncture but not sham acupuncture [STR concentration: *F*(4,12) = 19.16, *P* < 0.0001; SNpc concentration: *F*(4,12) = 14.95, *P* = 0.0001; PFC concentration: *F*(4,12) = 9.158, *P* = 0.0012; thalamus concentration: *F*(4,12) = 0.07692, *P* = 0.9879; Figure 2].

**FIGURE 3:**
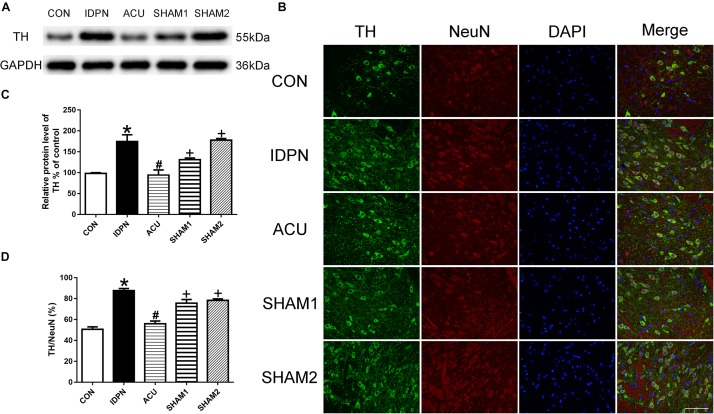
Effects of acupuncture on TH protein expression and the number of TH positive neurons in the SNpc. **(A)** Representative gel images showed the protein level of TH in the SNpc tissues obtained from the CON, IDPN, ACU, SHAM1, and SHAM2 groups. GAPDH was used as a loading control. **(B)** TH labeling (green); NeuN labeling (red); DAPI (blue). Scale bar, 100 μm. **(C)** Summary data showed effects of IDPN and acupuncture on the protein level of TH in the SNpc. **(D)** Summary graph shows the percentage of double-labeled TH and NeuN immunoreactivity in the total of NeuN-positive cells in the SNpc from different groups. Data are expressed as means ± SEM (*n* = 6 mice in each group). ^∗^*P* < 0.05, compared with the CON group; ^#^*P* < 0.05, compared with the IDPN group; ^+^*P* < 0.05, compared with the ACU group.

Since TH is considered as the rate-limiting enzyme in DA production (Dahoun et al., 2017), and the nerve terminals that release DA in the STR, SNpc, and PFC originate from dopaminergic neurons in the SNpc, we determined the effect of acupuncture on TH expression in the SNpc. As shown in Figure 3, the expression of TH in the IDPN group was significantly higher than that in the control group; acupuncture but not sham acupuncture significantly reduced the expression of TH [TH expression: *F*(4,8) = 19.16, *P* = 0.0004; Figures 3A,C]. The ratio of TH-positive neurons to neuronal nuclei (NeuN)-positive neurons was significantly increased in the IDPN group compared with the control group, which was significantly reversed by acupuncture but not sham acupuncture [TH/NeuN: *F*(4,8) = 33.41, *P* < 0.0001, Figures 3B,D]. These data demonstrated that acupuncture decreased the concentration of DA in the STR, SNpc, and PFC, and down-regulated TH expression and the distribution of TH positive neurons in the SNpc as well.

### Acupuncture Reduced D1R and D2R Expression in the STR and the SNpc

**FIGURE 4:**
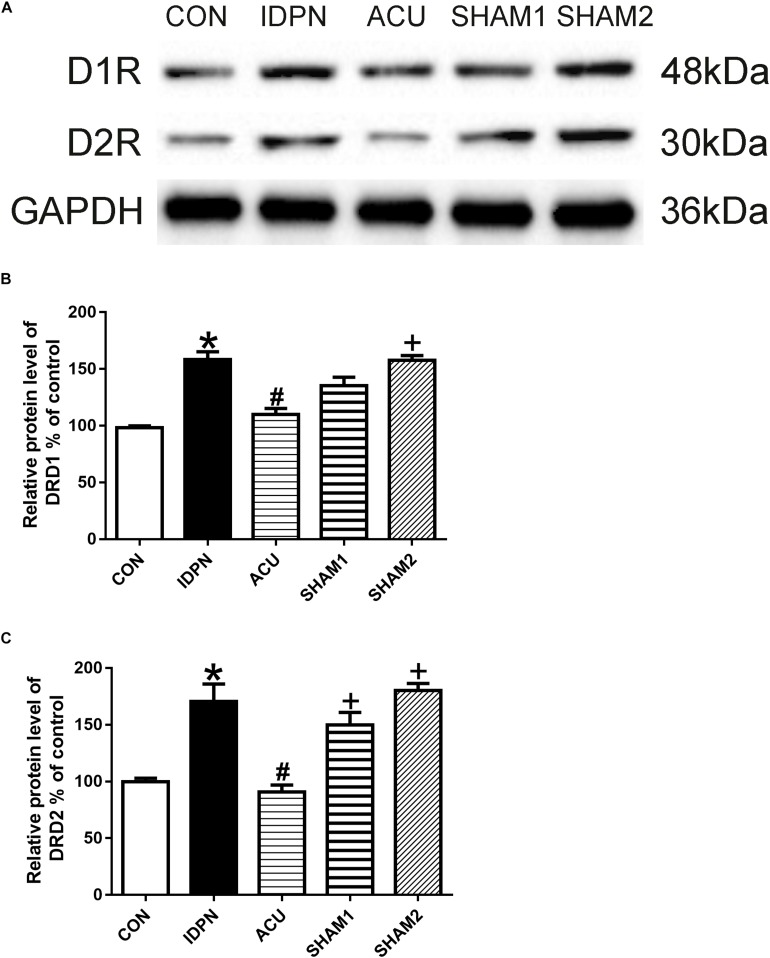
Effects of acupuncture on D1R and D2R protein expression in the STR. **(A)** Representative gel images showed the protein levels of D1R and D2R in the STR tissues obtained from the CON, IDPN, ACU, SHAM1, and SHAM2 groups. GAPDH was used as a loading control. **(B)** Summary data showed the effects of IDPN and acupuncture on protein level of D1R in the STR. **(C)** Summary data showed the effects of IDPN and acupuncture on protein level of D2R in the STR. Data are expressed as means ± SEM (*n* = 6 mice in each group). ^∗^*P* < 0.05, compared with the CON group; ^#^*P* < 0.05, compared with the IDPN group; ^+^*P* < 0.05, compared with the ACU group.

**FIGURE 5:**
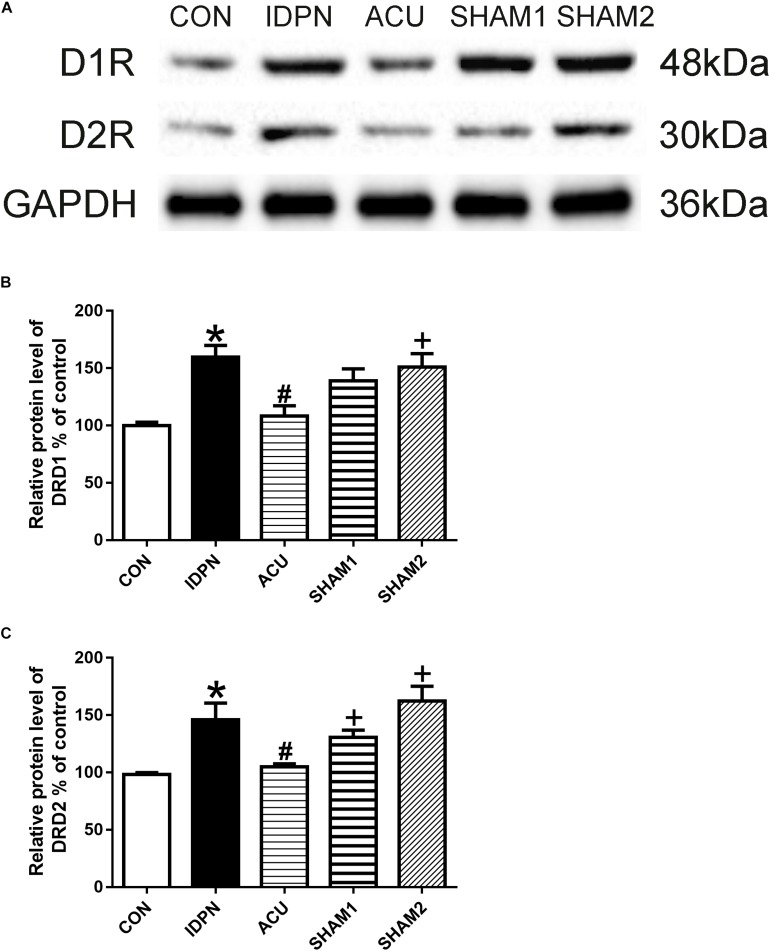
Effects of acupuncture on D1R and D2R protein expression in the SNpc. **(A)** Representative gel images showed the protein levels of D1R and D2R in the SNpc tissues obtained from the CON, IDPN, ACU, SHAM1, and SHAM2 groups. GAPDH was used as a loading control. **(B)** Summary data showed the effects of IDPN and acupuncture on protein level of D1R in the SNpc. **(C)** Summary data showed the effects of IDPN and acupuncture on protein level of D2R in the SNpc. Data are expressed as means ± SEM (*n* = 6 mice in each group). ^∗^*P* < 0.05, compared with the CON group; ^#^*P* < 0.05, compared with the IDPN group; ^+^*P* < 0.05, compared with the ACU group.

The STR controls movement execution through the balanced activity of the direct and indirect pathways in the D1R- and D2R-expressing medium spiny neurons (MSNs), respectively (Nespoli et al., 2018). We determined whether acupuncture affected D1R and D2R expression in the STR and SNpc. We found that D1R and D2R expression in the IDPN group was significantly higher than that in the control group. Compared with the IDPN group, the protein level of D1R and D2R in the STR was reduced in the acupuncture group but not in the sham acupuncture group [D1R expression: *F*(4,8) = 27.81, *P* < 0.0001; D2R expression: *F*(4,8) = 16.52, *P* = 0.0006; Figure 4] and SNpc [D1R expression: *F*(4,8) = 7.001, *P* = 0.0100; D2R expression: *F*(4,8) = 7.148, *P* = 0.0094; Figure 5].

**FIGURE 6:**
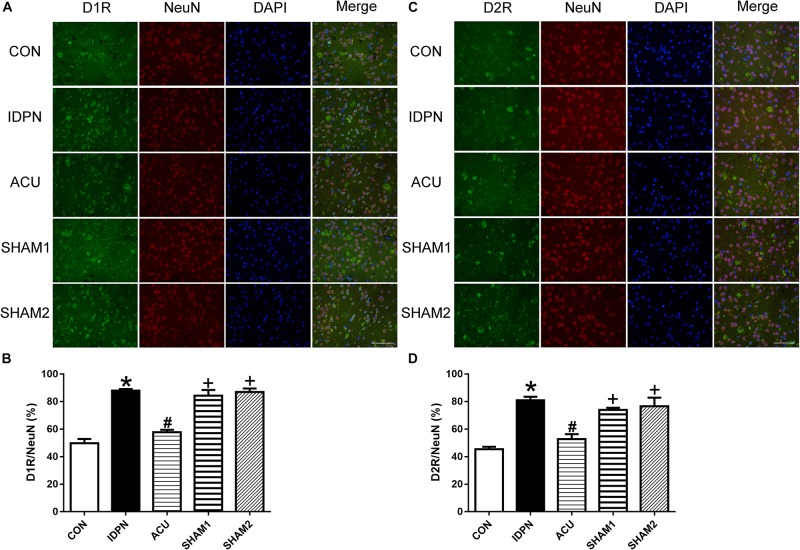
Effects of acupuncture on the number of D1R and D2R positive neurons in the STR. **(A)** D1R labeling (green); NeuN labeling (red); DAPI (blue). Scale bar, 100 μm. **(B)** Summary graph shows the percentage of double-labeled D1R and NeuN immunoreactivity in the total of NeuN-positive cells in the STR from different groups. **(C)** D2R labeling (green); NeuN labeling (red); DAPI (blue). Scale bar, 100 μm. **(D)** Summary graph shows the percentage of double-labeled D2R and NeuN immunoreactivity in the total of NeuN-positive cells in the STR from different groups. Data are expressed as means ± SEM (*n* = 6 mice in each group). ^∗^*P* < 0.05, compared with the CON group; ^#^*P* < 0.05, compared with the IDPN group; ^+^*P* < 0.05, compared with the ACU group.

**FIGURE 7:**
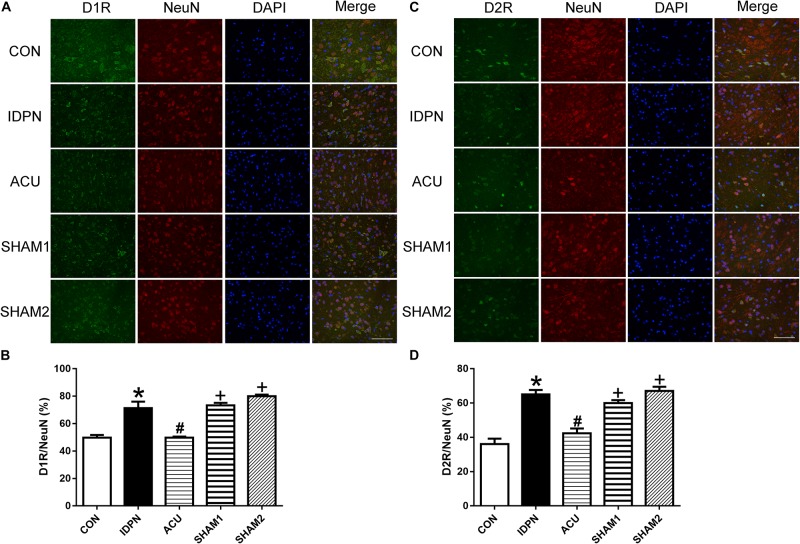
Effects of acupuncture on the number of D1R and D2R positive neurons in the SNpc. **(A)** D1R labeling (green); NeuN labeling (red); DAPI (blue). Scale bar, 100 μm. **(B)** Summary graph shows the percentage of double-labeled D1R and NeuN immunoreactivity in the total of NeuN-positive cells in the SNpc from different groups. **(C)** D2R labeling (green); NeuN labeling (red); DAPI (blue). Scale bar, 100 μm. **(D)** Summary graph shows the percentage of double-labeled D2R and NeuN immunoreactivity in the total of NeuN-positive cells in the SNpc from different groups. Data are expressed as means ± SEM (*n* = 6 mice in each group). ^∗^*P* < 0.05, compared with the CON group; ^#^*P* < 0.05, compared with the IDPN group; ^+^*P* < 0.05, compared with the ACU group.

We also used double-immunofluorescence labeling to determine the number of D1R and D2R positive neurons in the STR and SNpc. Consistent with the results of the protein expression, IDPN also significantly increased the ratio of D1R- and D2R-positive neurons to NeuN-positive neurons in the STR [D1R/NeuN: *F*(4,8) = 49.36, *P* < 0.0001; D2R/NeuN: *F*(4,8) = 67.58, *P* < 0.0001, Figure 6] and SNpc [D1R/NeuN: *F*(4,8) = 27.08, *P* = 0.0001; D2R/NeuN: *F*(4,8) = 34.24, *P* < 0.0001, Figure 7] compared with the control group, which was reversed by acupuncture but not sham acupuncture (*P* < 0.05). These results indicated that acupuncture significantly down-regulated the expression of D1R and D2R in the STR and SNpc.

### Acupuncture Reduced D1R but Not D2R Expression in the PFC, and Did Not Affect D1R and D2R Expression in the Thalamus

**FIGURE 8:**
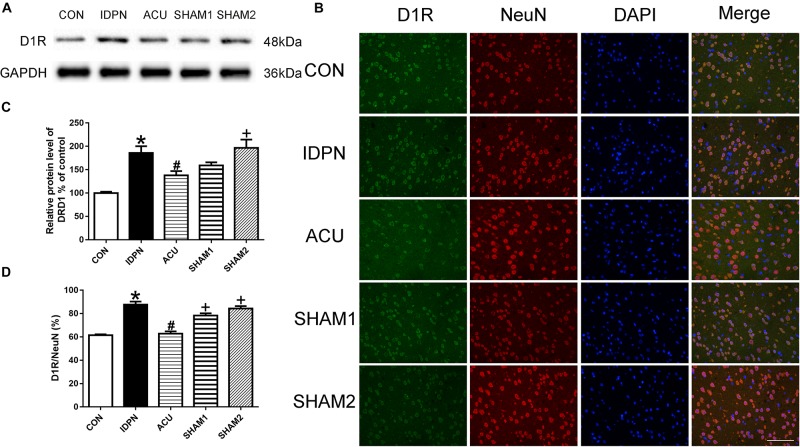
Effects of acupuncture on D1R protein expression and the number of D1R-positive neurons in the PFC. **(A)** Representative gel images showed the protein levels of D1R in the PFC tissues obtained from the CON, IDPN, ACU, SHAM1, and SHAM2 groups. GAPDH was used as a loading control. **(B)** D1R labeling (green); NeuN labeling (red); DAPI (blue). Scale bar, 100 μm. **(C)** Summary data showed the effects of IDPN and acupuncture on the protein level of D1R in the PFC. **(D)** Summary graph show the percentage of double-labeled D1R and NeuN immunoreactivity in the total of NeuN-positive cells in the PFC from different groups. Data are expressed as means ± SEM (*n* = 6 mice in each group). ^∗^*P* < 0.05, compared with the CON group; ^#^*P* < 0.05, compared with the IDPN group; ^+^*P* < 0.05, compared with the ACU group.

Since the PFC and the thalamus are both brain regions that are involved in TS (Baym et al., 2008; Steeves et al., 2010), we determined whether acupuncture affects the expression of D1R and D2R in the PFC and the thalamus. In the PFC, we found that the expression of D1R but not D2R in the IDPN group was significantly higher than that in the control group. Compared with the IDPN group, the expression of D1R but not D2R was reduced in the acupuncture group but not in the sham acupuncture group [D1R expression: *F*(4,8) = 10.81, *P* = 0.0026; D2R expression: *F*(4,8) = 0.2387, *P* = 0.9087; D1R/NeuN: *F*(4,8) = 37.66, *P* < 0.0001; D2R/NeuN: *F*(4,8) = 0.2257, *P* = 0.9165; Figure 8 and Supplementary Figure S2]. However, IDPN and acupuncture or sham acupuncture treatment had no influence on the expression of D1R and D2R in the thalamus [D1R expression: *F*(4,8) = 0.3889, *P* = 0.8112; D2R expression: *F*(4,8) = 0.7609, *P* = 0.5789; D1R/NeuN: *F*(4,8) = 0.1404, *P* = 0.9623; D2R/NeuN: *F*(4,8) = 0.4359, *P* = 0.7797; Supplementary Figure S3].

## Discussion

In this study, we used acupuncture treatment in a mouse model of TS for the first time, and we found that acupuncture alleviated the involuntary behavioral stereotypies in IDPN-induced TS model mice. In addition, acupuncture reduced the concentration of DA in the STR, SNpc, and PFC. It also reduced the expression of TH in the SNpc, D1R, and D2R in STR and SNpc, and D1R in the PFC. In conclusion, we demonstrated the effects of acupuncture on behavior and brain DA system in a mouse model of TS.

The time of acupuncture treatment varies from disease to disease. For example, chronic itch model mice are effective for acupuncture treatment for 9 days, while postherpetic neuralgia model mice need acupuncture treatment for 2 weeks to alleviate mechanical allodynia (Li et al., 2019). In a mouse model of TS, we determined that 4 weeks was the optimal treatment duration to alleviate the behavioral stereotypies. Consistently, the treatment duration of manual acupuncture on TS was around 30 days in clinical studies (Zhang, 2015). Therefore, the present study had great clinical significance for determining the duration of acupuncture treatment on TS.

Previous studies have shown that behavioral stereotypies are caused by the combined effects of excessive activity in motor pathways and reduced activity in cortico-striato-thalamo-cortical circuits (Mink, 2001; Church et al., 2009; Wang et al., 2011). The DA system is known to be an important modulator of striatum to process the cortical signals, which are carried by glutamatergic synapses on the principal neurons of the striatum-medium spiny neurons (STR-MSNs). Regulation of these neurons by DA is important for psychomotor functions ascribed to the basal ganglia, such as habit learning and the control of serial movement (Albin et al., 1989; Wickens et al., 2003; Schultz, 2006). The basal ganglia have been implicated in different processes that control action such as the control of movement parameters and processing of cognitive and emotional information from the environment (Buot and Yelnik, 2012). It may play a critical role in behavioral inhibition, mediated by the prefrontal, parietal, temporal, and cingulate cortices (Wang et al., 2007). In the present study, we found that acupuncture greatly alleviated rather than completely reversed behavioral stereotypies. This result might be attributed to the involvement of acupuncture in the regulation of the motor system of the basal ganglia. Our results confirmed that acupuncture could alleviate behavioral stereotypies, which might be a good theoretical guidance for the use of acupuncture in the clinical treatment of TS.

Striatum is the input nucleus of the basal ganglia, which is a crucial neuronal network for action selection and motor control (Thibault et al., 2013). The dopaminergic neurons of the SNpc, which project throughout the STR, are the main nuclei involved in DA synthesis in the brain (Lerner et al., 2012). Previous studies have shown that TS patients had greater neural activity within the SNpc than normal people did during the performance of spontaneous and involuntary behavioral stereotypies, reflecting an overactive nigrostriatal dopaminergic activity in TS patients (Baym et al., 2008; Davila et al., 2010; Wang et al., 2011). However, in contrast to healthy people, no significant DA release was noted in the thalamus of TS patients (Steeves et al., 2010). Our research showed that acupuncture decreased the concentration of DA in the STR, SNpc, and PFC in IDPN-induced TS model mice, which might contribute to the effect of acupuncture.

Tyrosine hydroxylase is the first enzyme in the catecholamine biosynthetic pathway. In the central nervous system, TH is expressed in areas with dopaminergic neurons including the SNpc (Kumer and Vrana, 1996). The capacity of TH neurons releasing DA will differ based on their capacity to accumulate vesicular DA, uptake extracellular DA, or be autoregulated by DA receptors (Morales and Root, 2014). Furthermore, a down-regulation of TH mRNA levels can lead to low levels of DA receptors expression (Bertotto et al., 2018). Consistently, in our study, an elevation of TH did not lead to a compensatory down-regulation of DA receptors in the IDPN group. We found that acupuncture reduced over-expression of both TH and D1R and D2R in the SNpc, which was consistent with the effect of acupuncture on behavioral stereotypies.

D1R and D2R are two major striatal DA receptors that can regulate synaptic plasticity. The main distinction between the two classes is that D1R activates adenyl cyclase, while D2R inhibits adenyl cyclase (Yoo et al., 2011). D1R signaling enhances dendritic excitability and glutamatergic signaling in striatonigral MSNs, whereas D2R signaling exerts the opposite effect (Surmeier et al., 2007). Striatonigral MSNs express high levels of D1R and D2R; striatonigral MSNs of the direct pathway project directly to neurons that interface between the basal ganglia and the rest of the brain, including neurons of the substantia nigra pars reticulata (SNr) and the internal segment of the globus pallidus (Surmeier et al., 2007). The indirect pathway of MSNs projects axons to the external segment of globus pallidus (Gerfen, 2006). D1R-expressing direct pathway MSNs mediate reinforcement of movement, while D2R-expressing indirect pathway MSNs mediate inhibition of movement (Bromberg-Martin et al., 2010; Lobo et al., 2010; Ferguson et al., 2011). IDPN induces the up-regulation of D1R and promotes the excitability of the direct pathway. Meanwhile, IDPN, which induces the up-regulation of D2R, may inhibit the excitability of the indirect pathway, leading to disinhibition of movement (Albin et al., 1989; Dreyer et al., 2010; Kravitz et al., 2012). As a result, activation of both D1R and D2R can promote movement. It suggested that acupuncture reduced the DA synthesis and its receptors expression in the STR, SNpc, and PFC, thereby inhibiting behavioral stereotypies. However, in the pathways of the basal ganglia, the PFC participates only in the direct pathway but not the indirect pathway (Frank et al., 2004; Hikida et al., 2010), which can explain why IDPN and acupuncture only affected the expression of D1R but not D2R in the PFC.

In summary, our findings provide new evidence that acupuncture inhibits behavioral stereotypies and regulates the DA system in the STR, SNpc, and PFC, which may reveal the mechanism of the therapeutic effect of acupuncture on TS. It provides a strong series of evidence for the application of acupuncture in treating TS in the clinic, so that TS patients could get safer and more effective treatment.

## Data Availability Statement

All datasets generated for this study are included in the manuscript/Supplementary Files.

## Ethics Statement

The animal study was reviewed and approved by the animal ethics committee of the Chinese Academy of Medical Sciences.

## Author Contributions

ML and WS conceived and designed the experiments. LL did most of the experiments and analyzed the data. XH, TH, and JC helped with the behavior test experiments. LY and HeZ helped with the western blotting and double-immunofluorescence labeling experiments. XY and HoZ helped with the DA test experiments. HL, HX, and SW helped with analyzing the data. LL, ML, and WS wrote the manuscript. All authors reviewed the manuscript.

## Conflict of Interest

The authors declare that the research was conducted in the absence of any commercial or financial relationships that could be construed as a potential conflict of interest.

## Supplementary Material

FIGURE S1 Experimental design timeline and effects of IDPN and acupuncture on body weight of mice. **(A)** All mice were fed for 1 week before building TS model. The CON group was intraperitoneally injected with saline (0.9%); the IDPN group, ACU, SHAM1, and SHAM2 groups were intraperitoneally injected with IDPN (350 mg/kg) at 10:00 a.m. once daily for seven consecutive days (days 1–7). In the ACU, SHAM1, and SHAM2 groups, the mice received acupuncture once every other day on days 8–36. All behavioral tests were carried out once every other week on days 0, 8, 15, 22, 29, and 36 after intraperitoneal injection or 1 h after acupuncture treatment. Brain tissues for Western blotting, immunofluorescence labeling, and fluorescence spectrophotometry were collected on day 37. **(B)** Summary data showed effects of IDPN and acupuncture on body weight of mice on days 0, 8, 15, 22, 29, and 36. Data are expressed as means ± SEM (*n* = 18 mice in each group). **(C)** Schematic diagram of the coordinates of acupoints “Baihui” (GV20) and “Yintang” (GV29). **(D)** Schematic diagram of acupuncture treatment. **(E)** Schematic diagram of the stereotyped behavior test.

FIGURE S2 Effects of acupuncture on D2R protein expression and the number of D2R-positive neurons in the PFC. **(A)** Representative gel images showed the protein levels of D2R in the PFC tissues obtained from the CON, IDPN, ACU, SHAM1, and SHAM2 groups. GAPDH was used as a loading control. **(B)** D2R labeling (green); NeuN labeling (red); DAPI (blue). Scale bar, 100 μm. **(C)** Summary data showed the effects of IDPN and acupuncture on protein level of D2R in the PFC. **(D)** Summary graph shows the percentage of double-labeled D2R and NeuN immunoreactivity in the total of NeuN-positive cells in the PFC from different groups. Data are expressed as means ± SEM (*n* = 6 mice in each group).

FIGURE S3 Effects of acupuncture on D1R and D2R protein expression and the number of D1R- and D2R-positive neurons in the thalamus. **(A)** Representative gel images showed the protein levels of D1R and D2R in the thalamus tissues obtained from the CON, IDPN, ACU, SHAM1, and SHAM2 groups. GAPDH was used as a loading control. **(B)** Summary data showed effects of IDPN and acupuncture on protein level of D1R in the thalamus. **(C)** Summary data showed effects of IDPN and acupuncture on the protein level of D2R in the thalamus. **(D)** D1R labeling (green); NeuN labeling (red); DAPI (blue). Scale bar, 100 μm. **(E)** D2R labeling (green); NeuN labeling (red); DAPI (blue). Scale bar, 100 μm. **(F)** Summary graph shows the percentage of double-labeled D1R and NeuN immunoreactivity in the total of NeuN-positive cells in the thalamus from different groups. **(G)** Summary graph shows the percentage of double-labeled D2R and NeuN immunoreactivity in the total of NeuN-positive cells in the thalamus from different groups. Data are expressed as means ± SEM (*n* = 6 mice in each group).

## References

[B1] Albin R. L. Young A. B. Penney J. B. (1989). The functional anatomy of basal ganglia disorders. *Trends Neurosci.* 12 366–375. 2479133 10.1016/0166-2236(89)90074-x

[B2] Baym C. L. Corbett B. A. Wright S. B. Bunge S. A. (2008). Neural correlates of tic severity and cognitive control in children with tourette syndrome. *Brain* 131 (Pt 1), 165–179. 10.1093/brain/awm278 18056159

[B3] Bertotto L. B. Richards J. Gan J. Volz D. C. Schlenk D. (2018). Effects of bifenthrin exposure on the estrogenic and dopaminergic pathways in zebrafish embryos and juveniles. *Environ. Toxicol. Chem.* 37 236–246. 10.1002/etc.3951 28815728

[B4] Bo A. Si L. Wang Y. Bao L. Yuan H. (2017). Mechanism of mongolian medical warm acupuncture in treating insomnia by regulating mir-101a in rats with insomnia. *Exp. Ther. Med.* 14 289–297. 10.3892/etm.2017.4452 28672928 PMC5488598

[B5] Brett P. M. Curtis D. Robertson M. M. Gurling H. M. (1995). The genetic susceptibility to gilles de la tourette syndrome in a large multiple affected british kindred: linkage analysis excludes a role for the genes coding for dopamine d1, d2, d3, d4, d5 receptors, dopamine beta hydroxylase, tyrosinase, and tyrosine hydroxylase. *Biol. Psychiatry* 37 533–540. 10.1016/0006-3223(94)00161-U 7619976

[B6] Bromberg-Martin E. S. Matsumoto M. Hikosaka O. (2010). Dopamine in motivational control: rewarding, aversive, and alerting. *Neuron* 68 815–834. 10.1016/j.neuron.2010.11.022 21144997 PMC3032992

[B7] Bronfeld M. Yael D. Belelovsky K. Bar-Gad I. (2013). Motor tics evoked by striatal disinhibition in the rat. *Front. Syst. Neurosci.* 7:50. 10.3389/fnsys.2013.00050 24065893 PMC3776161

[B8] Buot A. Yelnik J. (2012). Functional anatomy of the basal ganglia: limbic aspects. *Rev Neurol* 168 569–575. 10.1016/j.neurol.2012.06.015 22902172

[B9] Buse J. Schoenefeld K. Munchau A. Roessner V. (2013). Neuromodulation in tourette syndrome: dopamine and beyond. *Neurosci. Biobehav. Rev.* 37 1069–1084. 10.1016/j.neubiorev.2012.10.004 23085211

[B10] Butler I. J. Koslow S. H. Seifert W. E. Jr. Caprioli R. M. Singer H. S. (1979). Biogenic amine metabolism in Tourette syndrome. *Ann. Neurol.* 6 37–39. 10.1002/ana.410060109 292354

[B11] Cabyoglu M. T. Ergene N. Tan U. (2006). The mechanism of acupuncture and clinical applications. *Int. J. Neurosci.* 116 115–125. 10.1080/00207450500341472 16393878

[B12] Cath D. C. Gijsman H. J. Schoemaker R. C. van Griensven J. M. Troost N. van Kempen G. M. (1999). The effect of m-CPP on tics and obsessive-compulsive phenomena in gilles de la tourette syndrome. *Psychopharmacology* 144 137–143. 10.1007/s002130050986 10394994

[B13] Cavanna A. E. Selvini C. Termine C. Balottin U. Eddy C. M. (2012). Tolerability profile of clonidine in the treatment of adults with tourette syndrome. *Clin. Neuropharmacol.* 35 269–272. 10.1097/WNF.0b013e3182741c39 23123691

[B14] Cheng K. J. (2014). Neurobiological mechanisms of acupuncture for some common illnesses: a clinician’s perspective. *J. Acupunct. Meridian Stud.* 7 105–114. 10.1016/j.jams.2013.07.008 24929454

[B15] Chun L. S. Free R. B. Doyle T. B. Huang X. P. Rankin M. L. Sibley D. R. (2013). D1-D2 dopamine receptor synergy promotes calcium signaling via multiple mechanisms. *Mol. Pharmacol.* 84 190–200. 10.1124/mol.113.085175 23680635 PMC3716318

[B16] Church J. A. Fair D. A. Dosenbach N. U. Cohen A. L. Miezin F. M. Petersen S. E. (2009). Control networks in paediatric Tourette syndrome show immature and anomalous patterns of functional connectivity. *Brain* 132 (Pt 1), 225–238. 10.1093/brain/awn223 18952678 PMC2638693

[B17] Comings D. E. Gade R. Muhleman D. Sverd J. (1995). No association of a tyrosine hydroxylase gene tetranucleotide repeat polymorphism in autism. Tourette syndrome, or ADHD. *Biol. Psychiatry* 37 484–486. 10.1016/0006-3223(94)00311-P 7786965

[B18] Cui Y. Jin Z. Chen X. He Y. Liang X. Zheng Y. (2014). Abnormal baseline brain activity in drug-naive patients with Tourette syndrome: a resting-state fMRI study. *Front. Hum. Neurosci.* 7:913. 10.3389/fnhum.2013.00913 24427134 PMC3877773

[B19] Cui Y. H. Zheng Y. Jin Z. He Y. Chen X. Yu L. P. (2013). [Relationship between tic symptom severity and amplitude of low frequency fluctuation of resting-state functional magnetic resonance imaging of Tourette syndrome]. *Zhonghua Er Ke Za Zhi* 51 448–452. 24120064

[B20] Dahoun T. Trossbach S. V. Brandon N. J. Korth C. Howes O. D. (2017). The impact of disrupted-in-Schizophrenia 1 (DISC1) on the dopaminergic system: a systematic review. *Transl. Psychiatry* 7:e1015. 10.1038/tp.2016.282 28140405 PMC5299392

[B21] Davila G. Berthier M. L. Kulisevsky J. Asenjo B. Gomez J. Lara J. P. (2010). Structural abnormalities in the substantia nigra and neighbouring nuclei in tourette’s syndrome. *J. Neural Transm.* 117 481–488. 10.1007/s00702-010-0369-368 20131071

[B22] Deckersbach T. Chou T. Britton J. C. Carlson L. E. Reese H. E. Siev J. (2014). Neural correlates of behavior therapy for tourette’s disorder. *Psychiatry Res.* 224 269–274. 10.1016/j.pscychresns.2014.09.003 25444535 PMC4410879

[B23] Diamond B. I. Reyes M. G. Borison R. (1982). A new animal model for tourette syndrome. *Adv. Neurol.* 35 221–225. 6959491

[B24] Dreyer J. K. Herrik K. F. Berg R. W. Hounsgaard J. D. (2010). Influence of phasic and tonic dopamine release on receptor activation. *J. Neurosci.* 30 14273–14283. 10.1523/JNEUROSCI.1894-10.2010 20962248 PMC6634758

[B25] Du J. C. Chiu T. F. Lee K. M. Wu H. L. Yang Y. C. Hsu S. Y. (2010). Tourette syndrome in children: an updated review. *Pediatr. Neonatol.* 51 255–264. 10.1016/S1875-9572(10)60050-60052 20951354

[B26] Duo L. Hu L. Tian N. Cheng G. Wang H. Lin Z. (2018). TRPV1 gain-of-function mutation impairs pain and itch sensations in mice. *Mol. Pain* 14:1744806918762031. 10.1177/1744806918762031 29424270 PMC5846932

[B27] Eapen V. Ward P. Clarke R. (2014). Clonidine in Tourette syndrome and sensorimotor gating. *Psychiatry Res.* 215 494–496. 10.1016/j.psychres.2013.10.009 24210663

[B28] Eddy C. M. Cavanna A. E. Rickards H. E. Hansen P. C. (2016). Temporo-parietal dysfunction in Tourette syndrome: insights from an fMRI study of Theory of Mind. *J. Psychiatr. Res.* 81 102–111. 10.1016/j.jpsychires.2016.07.002 27424063

[B29] Egolf A. Coffey B. J. (2014). Current pharmacotherapeutic approaches for the treatment of tourette syndrome. *Drugs Today* 50 159–179. 10.1358/dot.2014.50.2.2097801 24619591

[B30] Faircloth A. (2015). Acupuncture: history from the yellow emperor to modern anesthesia practice. *AANA J.* 83 289–295. 26390748

[B31] Fang Z. C. (1993). The acupoints and acupuncture techniques in experimental rats. *J. Nanj. Railway Med. Coll.* 12 19–21.

[B32] Ferguson S. M. Eskenazi D. Ishikawa M. Wanat M. J. Phillips P. E. Dong Y. (2011). Transient neuronal inhibition reveals opposing roles of indirect and direct pathways in sensitization. *Nat. Neurosci.* 14 22–24. 10.1038/nn.2703 21131952 PMC3058296

[B33] Frank M. J. Seeberger L. C. O’Reilly R. C. (2004). By carrot or by stick: cognitive reinforcement learning in parkinsonism. *Science* 306 1940–1943. 10.1126/science.1102941 15528409

[B34] Gerfen C. R. (2006). Indirect-pathway neurons lose their spines in parkinson disease. *Nat. Neurosci.* 9 157–158. 10.1038/nn0206-157 16439979

[B35] Guo H. Ou-Yang Y. (2008). [Curative effect and possible mechanisms of topiramate in treatment of tourette syndrome in rats]. *Zhongguo Dang Dai Er Ke Za Zhi* 10 509–512. 18706175

[B36] Hikida T. Kimura K. Wada N. Funabiki K. Nakanishi S. (2010). Distinct roles of synaptic transmission in direct and indirect striatal pathways to reward and aversive behavior. *Neuron* 66 896–907. 10.1016/j.neuron.2010.05.011 20620875

[B37] Huang G. J. Edwards A. Tsai C. Y. Lee Y. S. Peng L. Era T. (2014). Ectopic cerebellar cell migration causes maldevelopment of Purkinje cells and abnormal motor behaviour in Cxcr4 null mice. *PLoS One* 9:e86471. 10.1371/journal.pone.0086471 24516532 PMC3917845

[B38] Israelashvili M. Bar-Gad I. (2015). Corticostriatal divergent function in determining the temporal and spatial properties of motor tics. *J. Neurosci.* 35 16340–16351. 10.1523/JNEUROSCI.2770-15.2015 26674861 PMC4679818

[B39] Jin W. Kim M. S. Jang E. Y. Lee J. Y. Lee J. G. Kim H. Y. (2018). Acupuncture reduces relapse to cocaine-seeking behavior via activation of GABA neurons in the ventral tegmental area. *Addict. Biol.* 23 165–181. 10.1111/adb.12499 28271626

[B40] Kawohl W. Bruhl A. Krowatschek G. Ketteler D. Herwig U. (2009). Functional magnetic resonance imaging of tics and tic suppression in gilles de la tourette syndrome. *World J. Biol. Psychiatry* 10 (4 Pt 2), 567–570. 10.1080/15622970802118356 18609432

[B41] Khan H. A. Alhomida A. S. Arif I. A. (2009). Neurovestibular toxicities of acrylonitrile and iminodipropionitrile in rats: a comparative evaluation of putative mechanisms and target sites. *Toxicol. Sci.* 109 124–131. 10.1093/toxsci/kfp043 19244277

[B42] Khan H. A. Ibrahim K. E. (2015). Pattern of neurobehavioral and organ-specific toxicities of beta, beta’-iminodipropionitrile in mice. *Arch. Med. Sci.* 11 1137–1144. 10.5114/aoms.2015.54871 26528360 PMC4624758

[B43] Kim N. J. Ryu Y. Lee B. H. Chang S. Fan Y. Gwak Y. S. (2018). Acupuncture inhibition of methamphetamine-induced behaviors, dopamine release and hyperthermia in the nucleus accumbens: mediation of group II mGluR. *Addict. Biol.* 24 206–217. 10.1111/adb.12587 29363229 PMC6890454

[B44] Kirshner N. (1957). Pathway of noradrenaline formation from DOPA. *J. Biol. Chem.* 226 821–825. 13438868

[B45] Kravitz A. V. Tye L. D. Kreitzer A. C. (2012). Distinct roles for direct and indirect pathway striatal neurons in reinforcement. *Nat. Neurosci.* 15 816–818. 10.1038/nn.3100 22544310 PMC3410042

[B46] Kumer S. C. Vrana K. E. (1996). Intricate regulation of tyrosine hydroxylase activity and gene expression. *J. Neurochem.* 67 443–462. 10.1046/j.1471-4159.1996.67020443.x 8764568

[B47] Lee M. H. (2017). A single case of tourette’s syndrome treated with traditional chinese medicine. *J. Acupunct. Meridian Stud.* 10 55–61. 10.1016/j.jams.2016.12.005 28254105

[B48] Lerner A. Bagic A. Simmons J. M. Mari Z. Bonne O. Xu B. (2012). Widespread abnormality of the gamma-aminobutyric acid-ergic system in Tourette syndrome. *Brain* 135 (Pt 6), 1926–1936. 10.1093/brain/aws104 22577221 PMC3359755

[B49] Li H. He Z. Su T. Ma Y. Lu S. Dai C. (2003). Protective action of recombinant neurturin on dopaminergic neurons in substantia nigra in a rhesus monkey model of parkinson’s disease. *Neurol. Res.* 25 263–267. 10.1179/016164103101201472 12739234

[B50] Li H. P. Su W. Shu Y. Yuan X. C. Lin L. X. Hou T. F. (2019). Electroacupuncture decreases Netrin-1-induced myelinated afferent fiber sprouting and neuropathic pain through mu-opioid receptors. *J. Pain Res.* 12 1259–1268. 10.2147/JPR.S191900 31118749 PMC6499485

[B51] Lin J. G. Chen C. J. Yang H. B. Chen Y. H. Hung S. Y. (2017). Electroacupuncture promotes recovery of motor function and reduces dopaminergic neuron degeneration in rodent models of parkinson’s disease. *Int. J. Mol. Sci.* 18:E1846. 10.3390/ijms18091846 28837077 PMC5618495

[B52] Lobo M. K. Covington H. E. III Chaudhury D. Friedman A. K. Sun H. Damez-Werno D. (2010). Cell type-specific loss of BDNF signaling mimics optogenetic control of cocaine reward. *Science* 330 385–390. 10.1126/science.1188472 20947769 PMC3011229

[B53] Ma S. Liu X. Y. Yu R. L. Chen L. J. (2006). [Clinical observation on acupuncture for treatment of Tourette’s syndrome]. *Zhongguo Zhen Jiu* 26 392–394. 16813178

[B54] Mallory M. J. Do A. Bublitz S. E. Veleber S. J. Bauer B. A. Bhagra A. (2016). Puncturing the myths of acupuncture. *J. Integr. Med.* 14 311–314. 10.1016/S2095-4964(16)60269-60268 27641603

[B55] Mink J. W. (2001). Basal ganglia dysfunction in tourette’s syndrome: a new hypothesis. *Pediatr. Neurol.* 25 190–198. 10.1016/s0887-8994(01)00262-4 11587872

[B56] Morales M. Root D. H. (2014). Glutamate neurons within the midbrain dopamine regions. *Neuroscience* 282 60–68. 10.1016/j.neuroscience.2014.05.032 24875175 PMC4397110

[B57] Nespoli E. Rizzo F. Boeckers T. Schulze U. Hengerer B. (2018). Altered dopaminergic regulation of the dorsal striatum is able to induce tic-like movements in juvenile rats. *PLoS One* 13:e0196515. 10.1371/journal.pone.0196515 29698507 PMC5919623

[B58] Neuner I. Kellermann T. Stocker T. Kircher T. Habel U. Shah J. N. (2010). Amygdala hypersensitivity in response to emotional faces in Tourette’s patients. *World J. Biol. Psychiatry* 11 858–872. 10.3109/15622975.2010.480984 20560820

[B59] Nomoto N. (2004). Inhibitory effect of free radical scavenger, MCI-186, in the increase of hydroxyl radical induced by iminodipropionitrile in rats. *J. Neurol. Sci.* 219 41–44. 10.1016/j.jns.2003.12.005 15050436

[B60] Ogawa N. Mizukawa K. Haba K. Sato H. (1990). Neurotransmitter and receptor alterations in the rat persistent dyskinesia model induced by iminodipropionitrile. *Eur. Neurol.* 30(Suppl. 1), 31–40. 10.1159/000117171 1968833

[B61] Parraga H. C. Harris K. M. Parraga K. L. Balen G. M. Cruz C. (2010). An overview of the treatment of Tourette’s disorder and tics. *J. Child Adolesc. Psychopharmacol.* 20 249–262. 10.1089/cap.2010.0027 20807063

[B62] Piccinni A. Veltri A. Marazziti D. Moroni I. Dell’Osso L. (2013). Effectiveness of a clozapine-aripiprazole combination in Tourette syndrome and bipolar spectrum disorder. *J. Neuropsychiatry Clin. Neurosci.* 25 E45. 10.1176/appi.neuropsych.12020032 23487230

[B63] Proietti Onori M. Ceci C. Laviola G. Macri S. (2014). A behavioural test battery to investigate tic-like symptoms, stereotypies, attentional capabilities, and spontaneous locomotion in different mouse strains. *Behav. Brain Res.* 267 95–105. 10.1016/j.bbr.2014.03.023 24675156

[B64] Qi C. Ji X. Zhang G. Kang Y. Huang Y. Cui R. (2018). Haloperidol ameliorates androgen-induced behavioral deficits in developing male rats. *J. Endocrinol.* 237 193–205. 10.1530/JOE-17-0642 29563235

[B65] Ren X. (2017). [Origin and thought on the philosophical ideas of acupuncture in chinese medicine]. *Zhongguo Zhen Jiu* 37 1323–1327. 10.13703/j.0255-2930.2017.12.018 29354999

[B66] Roessner V. Schoenefeld K. Buse J. Bender S. Ehrlich S. Munchau A. (2013). Pharmacological treatment of tic disorders and tourette Syndrome. *Neuropharmacology* 68 143–149. 10.1016/j.neuropharm.2012.05.043 22728760

[B67] Roth J. (2018). The colorful spectrum of tourette syndrome and its medical, surgical and behavioral therapies. *Parkinsonism. Relat. Disord.* 46(Suppl. 1), S75–S79. 10.1016/j.parkreldis.2017.08.004 28807495

[B68] Schultz W. (2006). Behavioral theories and the neurophysiology of reward. *Annu. Rev. Psychol.* 57 87–115. 10.1146/annurev.psych.56.091103.070229 16318590

[B69] Singer H. S. (2013). Motor control, habits, complex motor stereotypies, and tourette syndrome. *Ann. N. Y. Acad. Sci.* 1304 22–31. 10.1111/nyas.12281 24175737

[B70] Singer H. S. Butler I. J. Tune L. E. Seifert W. E. Jr. Coyle J. T. (1982). Dopaminergic dsyfunction in tourette syndrome. *Ann. Neurol.* 12 361–366. 10.1002/ana.410120408 6184010

[B71] Steeves T. D. Ko J. H. Kideckel D. M. Rusjan P. Houle S. Sandor P. (2010). Extrastriatal dopaminergic dysfunction in tourette syndrome. *Ann. Neurol.* 67 170–181. 10.1002/ana.21809 20225192

[B72] Surmeier D. J. Ding J. Day M. Wang Z. Shen W. (2007). D1 and D2 dopamine-receptor modulation of striatal glutamatergic signaling in striatal medium spiny neurons. *Trends Neurosci.* 30 228–235. 10.1016/j.tins.2007.03.008 17408758

[B73] Tang Y. Shang Q. Li W. Xu S. (2015). [Clinical controlled trial on infantile tourette syndrome treated with integrated therapy of acupuncture and medicine]. *Zhongguo Zhen Jiu* 35 141–144. 25854020

[B74] Tekin I. Roskoski R. Jr. Carkaci-Salli N. Vrana K. E. (2014). Complex molecular regulation of tyrosine hydroxylase. *J. Neural. Transm.* 121 1451–1481. 10.1007/s00702-014-1238-1237 24866693

[B75] Thibault D. Loustalot F. Fortin G. M. Bourque M. J. Trudeau L. E. (2013). Evaluation of D1 and D2 dopamine receptor segregation in the developing striatum using BAC transgenic mice. *PLoS One* 8:e67219. 10.1371/journal.pone.0067219 23843993 PMC3699584

[B76] Tinaz S. Belluscio B. A. Malone P. van der Veen J. W. Hallett M. Horovitz S. G. (2014). Role of the sensorimotor cortex in Tourette syndrome using multimodal imaging. *Hum. Brain Mapp.* 35 5834–5846. 10.1002/hbm.22588 25044024 PMC4776755

[B77] Vicario C. M. Gulisano M. Martino D. Rizzo R. (2016). Timing recalibration in childhood tourette syndrome associated with persistent pimozide treatment. *J. Neuropsychol.* 10 211–222. 10.1111/jnp.12064 25705969

[B78] Vinner E. Israelashvili M. Bar-Gad I. (2017). Prolonged striatal disinhibition as a chronic animal model of tic disorders. *J. Neurosci. Methods* 292 20–29. 10.1016/j.jneumeth.2017.03.003 28268105

[B79] Wang D. H. Li W. Liu X. F. Zhang J. M. Wang S. M. (2013). Chinese medicine formula “jian-pi-zhi-dong decoction” attenuates tourette syndrome via downregulating the expression of dopamine transporter in mice. *Evid Based Comp. Alternat Med.* 2013:385685. 10.1155/2013/385685 23431337 PMC3574653

[B80] Wang L. Lee D. Y. Bailey E. Hartlein J. M. Gado M. H. Miller M. I. (2007). Validity of large-deformation high dimensional brain mapping of the basal ganglia in adults with Tourette syndrome. *Psychiatry Res.* 154 181–190. 10.1016/j.pscychresns.2006.08.006 17289354 PMC2859464

[B81] Wang S. Qi F. Li J. Zhao L. Li A. (2012). Effects of Chinese herbal medicine ningdong granule on regulating dopamine (DA)/serotonin (5-TH) and gamma-amino butyric acid (GABA) in patients with Tourette syndrome. *Biosci. Trends* 6 212–218. 10.5582/bst.2012.v6.4.212 23006968

[B82] Wang Z. Maia T. V. Marsh R. Colibazzi T. Gerber A. Peterson B. S. (2011). The neural circuits that generate tics in Tourette’s syndrome. *Am. J. Psychiatry* 168 1326–1337. 10.1176/appi.ajp.2011.09111692 21955933 PMC4246702

[B83] Wickens J. R. Reynolds J. N. Hyland B. I. (2003). Neural mechanisms of reward-related motor learning. *Curr. Opin. Neurobiol.* 13 685–690. 10.1016/j.conb.2003.10.013 14662369

[B84] Wu C. H. Yuan X. C. Gao F. Li H. P. Cao J. Liu Y. S. (2016). Netrin-1 contributes to myelinated afferent fiber sprouting and neuropathic pain. *Mol. Neurobiol.* 53 5640–5651. 10.1007/s12035-015-9482-x 26482371

[B85] Wu L. Li H. Kang L. (1996). 156 cases of gilles de la tourette’s syndrome treated by acupuncture. *J. Tradit. Chin. Med.* 16 211–213. 9389122

[B86] Xiao L. Y. Yang J. W. Wang X. R. Ye Y. Yang N. N. Yan C. Q. (2018). Acupuncture rescues cognitive impairment and upregulates dopamine-beta-hydroxylase expression in chronic cerebral hypoperfusion rats. *Biomed Res. Int.* 2018:5423961. 10.1155/2018/5423961 30112399 PMC6077593

[B87] Xie H. Wang Z. Ji Y. Yin J. Yang W. H. Ren L. M. (2016). [Effects of salidroside on tic behavior of tourette syndrome model rats]. *Zhongguo Zhong Xi Yi Jie He Za Zhi* 36 90–93. 26955685

[B88] Yoo Y. C. Oh J. H. Kwon T. D. Lee Y. K. Bai S. J. (2011). Analgesic mechanism of electroacupuncture in an arthritic pain model of rats: a neurotransmitter study. *Yonsei Med. J.* 52 1016–1021. 10.3349/ymj.2011.52.6.1016 22028168 PMC3220264

[B89] Yu J. Ye Y. Liu J. Wang Y. Peng W. Liu Z. (2016). Acupuncture for tourette syndrome: a systematic review. *Evid Based Comp. Alternat Med.* 2016:1834646. 10.1155/2016/1834646 27725839 PMC5048029

[B90] Yuan X. C. Zhu B. Jing X. H. Xiong L. Z. Wu C. H. Gao F. (2018). Electroacupuncture potentiates cannabinoid receptor-mediated descending inhibitory control in a mouse model of knee osteoarthritis. *Front. Mol. Neurosci.* 11:112. 10.3389/fnmol.2018.00112 29681797 PMC5897736

[B91] Zebardast N. Crowley M. J. Bloch M. H. Mayes L. C. Wyk B. V. Leckman J. F. (2013). Brain mechanisms for prepulse inhibition in adults with Tourette syndrome: initial findings. *Psychiatry Res.* 214 33–41. 10.1016/j.pscychresns.2013.05.009 23916249 PMC3932431

[B92] Zhang F. Li A. (2015a). Dual ameliorative effects of Ningdong granule on dopamine in rat models of Tourette’s syndrome. *Sci. Rep.* 5:7731. 10.1038/srep07731 25592875 PMC4296291

[B93] Zhang F. Li A. (2015b). Dual regulating effects of gastrodin on extracellular dopamine concentration in rats models of Tourette’s syndrome. *Int. J. Neurosci.* 125 784–792. 10.3109/00207454.2014.971455 25271797

[B94] Zhang F. Li A. (2015c). Dual restoring effects of gastrodin on dopamine in rat models of tourette’s syndrome. *Neurosci. Lett.* 588 62–66. 10.1016/j.neulet.2014.12.051 25549540

[B95] Zhang H. X. (2015). The experience of the old chinese medicine zhang tangfa with scalp acupuncture in treating children with tourette syndrome. *Lishizhen Med. Materia Med. Res.* 26 2535–2537.

[B96] Zhang W. Yu W. Wang D. Wei L. Lee M. Wang S. (2014). Effect of “jian-pi-zhi-dong decoction” on gamma-aminobutyric Acid in a mouse model of tourette syndrome. *Evid Based Comp. Alternat Med.* 2014:407509. 10.1155/2014/407509 24812567 PMC4000640

[B97] Zhao L. Qi F. Zhang F. Wang Z. Mu L. Wang Y. (2015). Dual regulating effect of Ningdong granule on extracellular dopamine content of two kinds of Tourette’s syndrome rat models. *Biosci. Trends* 9 245–251. 10.5582/bst.2015.01088 26248644

[B98] Zhu B. C. Shi-fen X. Shan Y. H. (2009). [Clinical study on scalp acupuncture with long needle-retained duration for treatment of Tourette syndrome]. *Zhongguo Zhen Jiu* 29 115–118. 19391534

[B99] Zhu Y. Zhang J. Zeng Y. (2012). Overview of tyrosine hydroxylase in parkinson’s disease. *CNS Neurol Disord. Drug Targets* 11 350–358. 10.2174/187152712800792901 22483316

