# Supplementary figures and images for: Effects of Acupuncture on Behavioral Stereotypies and Brain Dopamine System in Mice as a Model of Tourette Syndrome
Source: Front Behav Neurosci. 2019 Oct 15;13:239. doi: 10.3389/fnbeh.2019.00239 (PMC6803462; doi:10.3389/fnbeh.2019.00239)

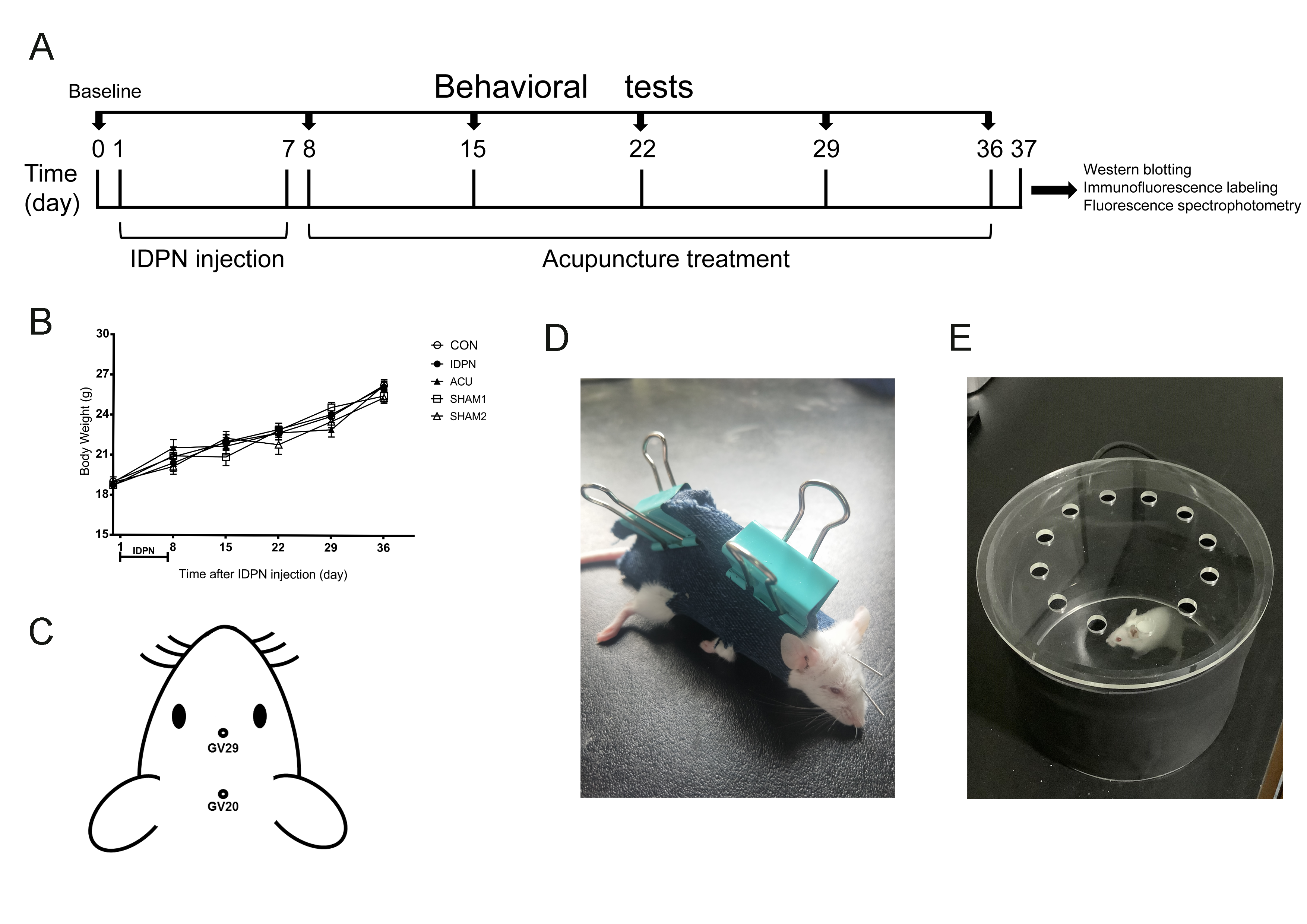

Supplement: FIGURE S1 — Experimental design timeline and effects of IDPN and acupuncture on body weight of mice. (A) All mice were fed for 1 week before building TS model. The CON group was intraperitoneally injected with saline (0.9%); the IDPN group, ACU, SHAM1, and SHAM2 groups were intraperitoneally injected with IDPN (350 mg/kg) at 10:00 a.m. once daily for seven consecutive days (days 1–7). In the ACU, SHAM1, and SHAM2 groups, the mice received acupuncture once every other day on days 8–36. All behavioral tests were carried out once every other week on days 0, 8, 15, 22, 29, and 36 after intraperitoneal injection or 1 h after acupuncture treatment. Brain tissues for Western blotting, immunofluorescence labeling, and fluorescence spectrophotometry were collected on day 37. (B) Summary data showed effects of IDPN and acupuncture on body weight of mice on days 0, 8, 15, 22, 29, and 36. Data are expressed as means ± SEM (n = 18 mice in each group). (C) Schematic diagram of the coordinates of acupoints “Baihui” (GV20) and “Yintang” (GV29). (D) Schematic diagram of acupuncture treatment. (E) Schematic diagram of the stereotyped behavior test. [file Image_1.TIF]

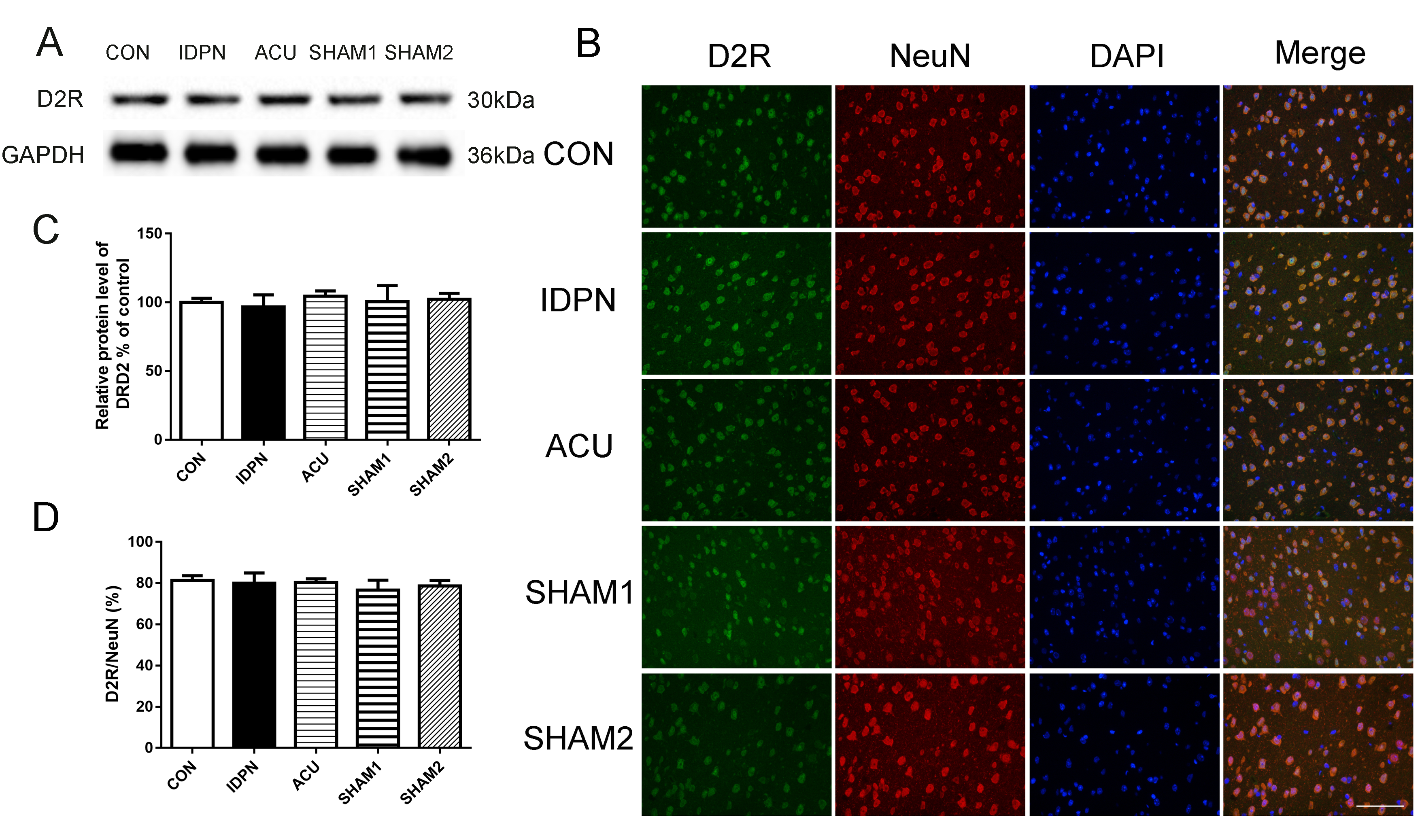

Supplement: FIGURE S2 — Effects of acupuncture on D2R protein expression and the number of D2R-positive neurons in the PFC. (A) Representative gel images showed the protein levels of D2R in the PFC tissues obtained from the CON, IDPN, ACU, SHAM1, and SHAM2 groups. GAPDH was used as a loading control. (B) D2R labeling (green); NeuN labeling (red); DAPI (blue). Scale bar, 100 μm. (C) Summary data showed the effects of IDPN and acupuncture on protein level of D2R in the PFC. (D) Summary graph shows the percentage of double-labeled D2R and NeuN immunoreactivity in the total of NeuN-positive cells in the PFC from different groups. Data are expressed as means ± SEM (n = 6 mice in each group). [file Image_2.TIF]

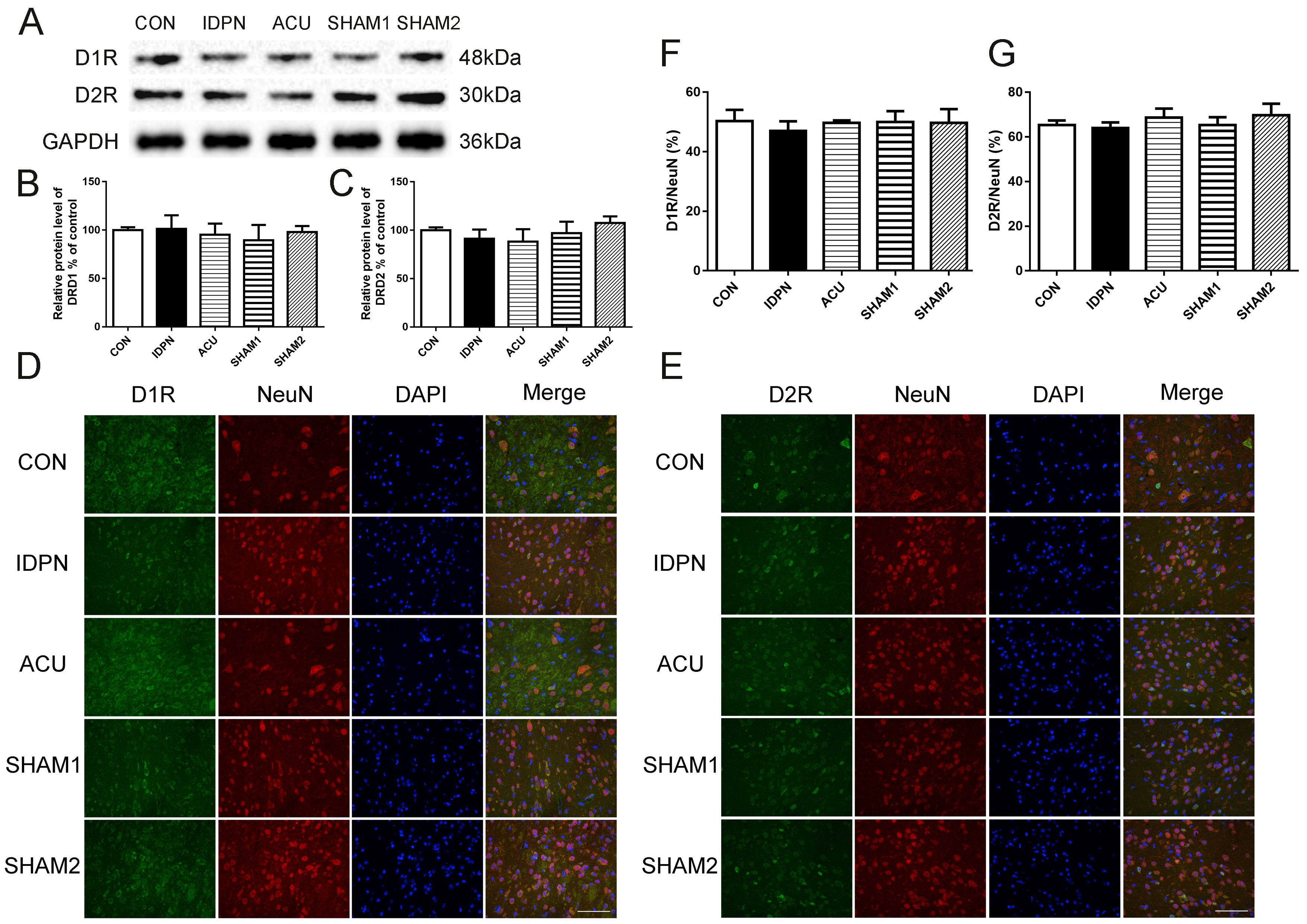

Supplement: FIGURE S3 — Effects of acupuncture on D1R and D2R protein expression and the number of D1R- and D2R-positive neurons in the thalamus. (A) Representative gel images showed the protein levels of D1R and D2R in the thalamus tissues obtained from the CON, IDPN, ACU, SHAM1, and SHAM2 groups. GAPDH was used as a loading control. (B) Summary data showed effects of IDPN and acupuncture on protein level of D1R in the thalamus. (C) Summary data showed effects of IDPN and acupuncture on the protein level of D2R in the thalamus. (D) D1R labeling (green); NeuN labeling (red); DAPI (blue). Scale bar, 100 μm. (E) D2R labeling (green); NeuN labeling (red); DAPI (blue). Scale bar, 100 μm. (F) Summary graph shows the percentage of double-labeled D1R and NeuN immunoreactivity in the total of NeuN-positive cells in the thalamus from different groups. (G) Summary graph shows the percentage of double-labeled D2R and NeuN immunoreactivity in the total of NeuN-positive cells in the thalamus from different groups. Data are expressed as means ± SEM (n = 6 mice in each group). [file Image_3.TIF]
